# A Case of Peritonitis, Consequent upon Bilious Fever, Terminating in a Copious Secretion of Pus into the Cavity of the Abdomen

**Published:** 1841-02

**Authors:** A. H. Buchanan

**Affiliations:** Columbia, Tennessee


ART. V.—*A case of Peritonitis, consequent upon Bilious Fever, terminating in a copious secretion of pus into the cavity of the Abdomen.* By. A. H. BUCHANAN, M. D., of Columbia, Tennessee.

ON the 10th of May, 1837, I was requested to visit a mulatto girl, aged about eight years, who had been seized forty-eight hours previously with bilious fever. About three years previously she suffered severely from scarlet fever, from the effects of which, however, she had entirely recovered, although of a delicate frame and rather feeble constitution. I found her laboring under the ordinary symptoms of bilious fever. By the use of the lancet, mercurial purges and mild febrifuge drinks, the febrile symptoms disappeared, and on the tenth day, believing her to be entirely convalescent, no further attention was given to the case. But in six days I was again requested to visit her, and found her suffering with severe pain in the abdomen and sick stomach, which was thought by her mother to be colic. The abdomen was much distended and very tender to the touch. On inquiring into the particulars of her relapse, I came to the conclusion that this attack was produced by exposure to cold. She was bled, put into a warm bath, and ordered a dose of calomel, to be followed in six hours by castor oil; after which, a large blistering plaster was applied over the abdomen, and mucilaginous drinks directed. For two weeks her bowels were kept open by the occasional use of calomel and laxatives, with a gradual amendment of her symptoms. The tenderness and soreness of the abdomen had nearly subsided, and she was able to sit up; there was some return of appetite; but the pulse was quick and irritable, and she suffered from hectic fever, while the abdomen became more and more distended, and emaciation progressed. At this time, the end of five weeks from her first attack, she had nearly all the symptoms of ascites: the abdomen was largely distended, and fluctuation upon percussion was very perceptible; the urine was scanty and micturition painful. Believing that there was a serous effusion into the cavity of the abdomen, I put her upon the use of hydragogue cathartics for three or four days, but without the least benefit. I therefore determined to tap her, and accordingly, in the presence of Messrs. Brown and Pillow, students of medicine, and others, I introduced the trocar at the usual point in the linea alba, about two inches below the umbilicus, when, upon withdrawing the stilet, to my antonishment pus flowed through the canula, until about half a gallon was discharged. The matter had all the external appearances of pus discharged from an abscess of the cellular tissue, and had no disagreeable odour. The child bore the operation well, and for five or six days improved in strength and spirits; but at the end of ten days the abdomen was as large as ever, and the matter pressed with great force at the point where the operation was performed, as if it would burst through. The trocar was, therefore, again introduced at the same point, and as much pus as at first was evacuated, the last ounce or two of which was thicker and of a greenish cast. The child now gradually improved in flesh and strength for eight or ten days, when the abdomen becoming again distended, it was thought advisable to let the matter discharge itself through the umbilicus where it now pointed. A poultice was ordered to be applied over the apex of the tumor, and in a few days the matter burst through at this point, and continued to discharge, more or less, for about three weeks, when the child appeared perfectly well, with the abdomen of the natural size. She has continued well, and grown rapidly, up to this date.

*Observations.*—When peritoneal inflammation does not terminate in resolution, the effusion of serum or of lymph is much more frequently the consequence, than that of so large an amount of purulent matter as occurred in this case. Such cases, indeed, are of very rare occurrence; although similar ones are mentioned by different writers on peritonitis. Baillie, in his Morbid Anatomy, observes, that "in some instances instead of serum a large quantity of pus is found;" and it is remarked by all pathological writers, that the effusion of pus is an occasional termination of peritonitis. M. Gase says, that he has known patients recover in whom the purulent matter escaped by the umbilicus.* M. Scoutetten also mentions a case; two cases of puerperal peritonitis which terminated in suppuration, and discharge by the umbilicus, are

* Quoted by Stokes in Cyclopedia of Prac. Med.: vol. 3, p. 294.

recorded by Gordon. Similar cases by different authors are on record; but it is remarked by Dr. Stokes that "It is doubtful when pus and lymph are effused in any great quantity, if they are ever absorbed, and such cases generally terminate fatally, or pass into the chronic form of the disease."* In the case I have related it is highly probable the disease would have terminated fatally, had not the matter been evacuated by tapping; for the child was extremely emaciated, and suffered much from hectic fever. Had I continued the use of the hydragogue cathartics the child would certainly have sunk; and had I delayed to the last the use of the instrument in the hope that the effused fluid would be absorbed, as is generally inculcated by writers on ascites, this child would most probably have perished. I consider it fortunate, therefore, that the trocar was introduced, and although I was not prepared to witness the evacuation of pus, I was immediately sensible on its appearance of an indescribable difference in the sensation produced on percussion in this case, and previous cases of ascites which I have met with. There was a greater weight, and a want of that perfect fluidity, which is present in the latter affection.

*October* 15, 1840.

---

### Art. VI.—*Observations on a peculiar form of Bilious Fever.*

By John Hardin, M. D., of Greensburg, Ky.

In an essay on Congestive Fever, published in the first Number of the Louisville Journal of Medicine and Surgery, two cases were reported on account of a peculiar character-

---

* Stokes in Cyclopedia of Prac. Med.: vol. 3. p. 294.

